# Ten Years of Animal Tuberculosis Monitoring in Free-Living European Bison (*Bison bonasus*) in Poland

**DOI:** 10.3390/ani13071205

**Published:** 2023-03-30

**Authors:** Monika Krajewska-Wędzina, Michał K. Krzysiak, Małgorzata Bruczyńska, Blanka Orłowska, Anna Didkowska, Łukasz Radulski, Jan Wiśniewski, Wanda Olech, Aneta Nowakiewicz, Mirosław Welz, Stanisław Kaczor, Marcin Weiner, Krzysztof Anusz

**Affiliations:** 1 National Veterinary Research Institute, 24-100 Puławy, Poland; 2 Department of Veterinary Microbiology, Faculty of Veterinary Medicine, Institute of Preclinical Veterinary Sciences, University of Life Sciences, 20-033 Lublin, Poland; 3 Białowieża National Park, 17-230 Białowieża, Poland; 4 Institute of Forest Sciences, Faculty of Civil Engineering and Environmental Sciences, Białystok University of Technology, 15-351 Białystok, Poland; 5 Department of Food Hygiene and Public Health Protection, Institute of Veterinary Medicine, Warsaw University of Life Sciences (SGGW), Nowoursynowska 159, 02-776 Warsaw, Poland; 6 County Veterinary Inspectorate, Orężna 9, 05-501 Piaseczno, Poland; 7 County Veterinary Inspectorate, C.K. Norwida 17, 24-100 Puławy, Poland; 8 Department of Animal Genetics and Conservation, Warsaw University of Life Sciences—SGGW, Ciszewskiego 8, 02-786 Warsaw, Poland; 9 Provincial Veterinary Inspectorate, Piotra Ścigiennego 6a, 38-400 Krosno, Poland; 10 County Veterinary Inspectorate, Młynarska 45, 38-500 Sanok, Poland; 11 Pope John Paul II State School of Higher Education, 21-500 Biała Podlaska, Poland

**Keywords:** European bison, Poland, *Mycobacterium bovis*, SB2806, MTBC, *Mycobacterium avium* spp. *hominissuis*

## Abstract

**Simple Summary:**

Between 1996–2012, two outbreaks of animal tuberculosis were noted in the free-living European bison population in the Bieszczady Mountains, Poland. As the European bison is an endangered species and is particularly susceptible to tuberculosis, not to mention a national icon, the decision was made to test all deceased bison for TB in Poland. The screened bison were obtained by elimination due to poor health or natural death. A total of 159 animals have been examined over the last 10 years. *Mycobacterium bovis* and *Mycobacterium avium* spp. *hominisuis* were identified in two different herds. The isolation of *M. bovis* from European bison is the first case described in Poland. Previously, the only causative agent of tuberculosis identified in European bison in Poland, both in the wild and in captive herds, was *Mycobacterium caprae*.

**Abstract:**

In the period 1996–2012, two outbreaks of animal tuberculosis were noted in the population of free-living European bison (*Bison bonasus caucasicus*) in the Bieszczady Mountains, Southern Poland. As the European bison is an endangered species and particularly susceptible to tuberculosis, not to mention a national icon, the decision was made to test all deceased bison for TB in Poland. The screened bison were obtained by elimination due to poor health or natural death. A total of 159 European bison have been examined over the last 10 years. The individuals came from four regions of Poland (Białowieża Forest, Bieszczady Mountains, Borecka Forest, Knyszyńska Forest), not only from the area where tuberculosis is still endemic. *Mycobacterium bovis* and *Mycobacterium avium* spp. *hominisuis* were identified in two different herds. The isolation of *M. bovis* from European bison was the first case described in Poland. So far, the only causative agent of tuberculosis identified in European bison in Poland, both in the wild and in captive herds, was *Mycobacterium caprae*. The isolated *M. bovis* spoligotype has not previously been registered in international spoligotype databases so far. The obtained results highlight the need to monitor TB in European bison in Poland.

## 1. Introduction

The wisent, or European bison (*Bison bonasus*), is the largest land mammal in Europe. The average body weight of males is 700 kg and for females is 450 kg. Its status is defined by the International Union for Conservation of Nature (IUCN) as “Near Threatened”; until 2020, the wisent was classified as “Vulnerable” [1]. Its population has tripled in the world in the last 20 years, growing from 2864 individuals, 717 in Poland, in 2000, to 9111 individuals globally in 2020 [2]. The population continues to grow. For example, the free-living population of bison increased from 456 in 2008 to 779 at the beginning of 2022 in the Białowieża Forest, and from 256 in 2013 to 729 in the Bieszczady Mountains [3]. Currently, the main threats to the European bison in Poland are its excessive density in a given area and the fact that it constitutes a small population with a low level of genetic diversity, which may reduce fertility and survival rate [4]. These factors may also contribute to an increased risk of infectious diseases such as animal tuberculosis (*Mycobacteriu bovis* and *Mycobacterium caprae*), pasteurellosis (*Pasteurella multocida*), or thelasiosis (*Thelazia gulosa*, *Thelazia skrjabini*) [5,6,7,8]. 

Hence, the health of the Polish wisent population has been under considerable scrutiny for a number of years [9]. A number of breeding committees act as advisory bodies to the custodians of bison herds. These commissions are appointed by the Regional Directorates of State Forests or, as in the case of the Białowieża population, by the Regional Directorate for Environmental Protection. Breeding committees are appointed by the orders of the directors of these units. They meet as needed and recommend actions needed in a specific herd, including those related to health and possible elimination of animals, but also discuss other elements of population management.

Most of the wisent populations in Poland have been under active surveillance for endemic and emerging infectious health threats since 2011 [9,10,11]; most of the studies have been aimed at epidemiological analysis and risk factor assessment as an element of the health protection strategy and preventive measures [9,12]. For over four years, no cases of TB have been recorded in European bison in Poland. The European bison has also been studied as a potential reservoir or indicator species in the transmission of diseases other than *Mycobacterium* spp. zoonotic agents [6,13,14,15]. A new challenge in the European bison protection strategy involves the increasing occurrence of pasteurellosis in free-living European bison, which is superseding the clinically-similar TB [6].

The aim of the study was active surveillance for TB in free-living European bison in Poland. As tuberculosis has already been recorded in European bison in Poland, both in captive and free-range herds [5], the results of this study may primarily contribute to faster epidemiological actions to prevent the spread of tuberculosis in the environment.

## 2. Materials and Methods

### 2.1. Animals and Samples

**Figure 1:**
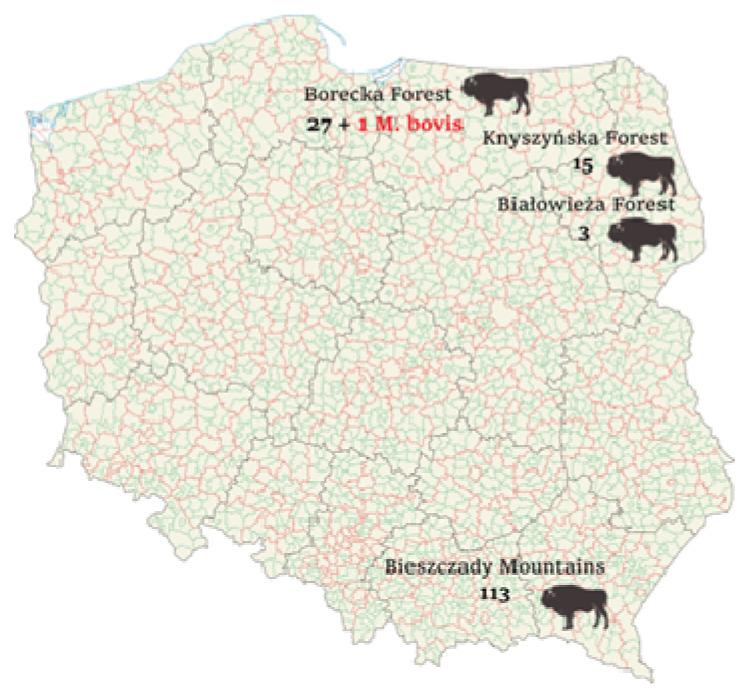
Location of the free-living herds from which the animals were tested.

In the years 2012–2021, 159 European bison (82 males, 67 females, undetermined in 10 cases) were tested post-mortem for TB. The examined wisent ranged in age from 5 months to 22 years (x = 10.82/years). They were derived from four free-living herds in different areas of Poland: Bieszczady Mountains, Borecka Forest, Knyszynska Forest, and Białowieza Forest (Figure 1). 

All tissues for examination were collected post-mortem. The most common cause of death was sanitary shooting and natural death. In one case, the cause was a traffic accident and there was one drowning of a very young individual in a well. In 10 cases, it was difficult to determine the final cause of death due to the fact that only the head and limbs were preserved and the rest of the body had been eaten by other forest game species such as wolves, wild boars, and foxes.

Retropharyngeal, tracheobronchial, mediastinal lymph nodes, a lung, and a liver segment were collected from animals with preserved whole bodies (n = 149). In the remaining 10 cases, the person collecting the samples dissected retropharyngeal and inguinal lymph nodes (n = 10).

The samples were sent to the National Veterinary Research Institute (NVRI) in Pulawy (Poland) to evaluate the prevalence of TB and mycobacteriosis. 

### 2.2. Mycobacterial Isolation

The first stage of the study was to examine the tissues to locate lesions suggestive of TB. The culture method performed in the NVRI was previously described by Radulski et al. [16]. The prepared sediment was plated on Stonebrink and Petragniani media (Media Department, NVRI, Pulawy, Poland) in triplicate and then incubated at 37 °C ± 2 °C [17]. After a two-week incubation period, the media were inspected once a week. 

### 2.3. DNA Extraction and Multiplex PCR

DNA isolation was performed using Genomic Mini AX Bacteria (A&A Biotechnology, Gdańsk, Poland) following the manufacturer’s instructions (https://www.aabiot.com/pobierz?code=15de0a1fe3fd35e7688cf0fe68a91af304ece5d2; accessed on 4 March 2023). To determine whether a given strain belonged to tuberculous or nontuberculous mycobacteria, multiplex PCR was performed (Table 1, Table 2 and Table 3), as previously described [16]. In this method, two pairs of primers were used, which allowed for the simultaneous amplification of two different DNA fragments [18]. The primers were constructed by the European Union Reference Laboratory for bovine tuberculosis in Madrid and synthesized by Eurofins Genomics company (Eurofins Genomics, Ebersberg, Germany).

### 2.4. Strain Species Identification

The strain was identified at the species level using the Genotype Mycobacterium CM and Genotype Mycobacterium MTBC tests (Hain Lifescience, Nehren, Germany) in accordance with the manufacturer’s protocols [19,20]. For nontuberculous mycobacteria strains, the strain was subjected to further testing to confirm the previous findings and determine the subspecies.

The results of the Hain Lifescience CM test were confirmed by MALDI-TOF mass spectrometry with a Bruker Biotyper System (Bruker, Billerica, MA, USA). For this purpose, an amount of bacterial biomass corresponding to two full volumes of a calibrated 1 µL inoculation loop was added to a test tube containing 50 µL of trifluoroacetic acid (TFA). The prepared sample was incubated for 30 min at room temperature and then diluted tenfold by adding 450 µL of sterile distilled water. Following this, 1 µL of the diluted mixture was applied to a MALDI plate. After the applied sample was dry, 1 µL of the matrix was added and analyzed.

Spoligotyping was performed on a single MTBC isolate cultured from retropharyngeal, tracheobronchial, and mediastinal lymph nodes from infected wisent. The spoligotyping tests were accomplished using a commercial genotyping kit (Gentaur molecular products, Kampenhout, Belgium) according to the manufacturer’s recommendations (https://gentaur-spain.com/wp-content/uploads/2015/03/Spoligotyping-Manual.pdf, accessed on 4 March 2023). The result was compared with patterns from international spoligotype databases, the Spol DB4 database (http://www.pasteur-guadeloupe.fr:8081/SITVIT_ONLINE/; accessed on 4 March 2023), and the Mbovis.org database [21].

## 3. Results

The gross inspection of the lymph nodes and liver and lung segments found them to be physiologically normal (n = 149). The exceptions were tissues from 10 carcasses, which were in the stage of decomposition by anaerobic bacteria (n = 10). No lesions indicating infection with bacteria of the genus *Mycobacterium* were present in any examined tissue samples (retropharyngeal, tracheobronchial, mediastinal lymph nodes, a lung, and a liver segment). In the culture study, two strains were obtained from two different European bison from two different herds. In one case, strain 2016 was obtained only on Stonebrink medium. In the second case, strain 2017, the growth was visible on both, Stonebrink and Petragnani substrates.

The use of multiplex PCR made it possible to distinguish between tuberculous and non-tuberculous strains. The nontuberculous mycobacteria genome contains only the 16S rRNA gene sequence, while the tuberculous mycobacteria genome contains both the 16S rRNA gene and the MPB70 protein gene. Therefore, in the first case we will get 1 DNA band, and in the second two. In strain 2016, only one DNA band at 1030 bp was obtained, which clearly indicated that it is a nontuberculous mycobacteria strain. In strain 2017, two DNA bands were obtained at 1030 bp and 372 bp, confirming that it belonged to the MTBC.

**Figure 2:**
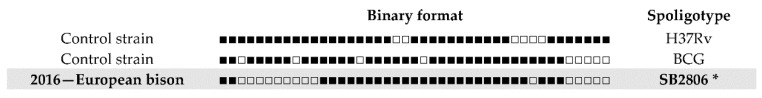
Spoligotyping results. Hybridization patterns (spoligotypes) of amplified mycobacterial DNAs of a strain isolated from European bison (* Assigned by www.Mbovis.org, accessed on 4 March 2023).

**Figure 3:**
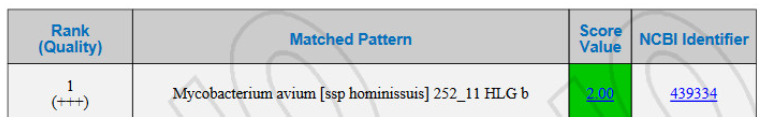
MALDI-TOF results. A score value ≥ 2.0 represents high-confidence identification.

To identify the species, the first strain was then subjected to the GenoType *Mycobacterium* MTBC test and the second to the GenoType *Mycobacterium* CM test. In the case of the first (i.e., tuberculous) strain, a distinct stripe was obtained at positions 4, 7, 9, and 10 in the GenoType MTBC confirming the presence of *M. bovis*. Genetic analysis showed that the 2016 strain was classified as *Mycobacterium bovis* spoligotype SB2806, not registered in databases so far (Figure 2). The results of the GenoType Mycobacterium CM test confirmed the presence of *M. avium* in the European bison tissue. The result was confirmed by MALDI-TOF: The obtained mass-to-charge ratio of the cellular proteins was automatically compared with all those present in the Bruker database, thus confirming *M. avium* ssp. *hominissuis* (Figure 3).

## 4. Discussion

Since early 2012, our laboratory has received tissue samples from the Borki Forest Inspectorate for analysis as part of routine TB check-ups. The forest inspectorate is the basic, independent organizational unit of the State Forests, operating on the basis of the Act on Forests. It is subordinate to the Regional Directorate of State Forests, which supervises and coordinates activities in its area. In total, the material was provided from 28 European bison (Figure 1). The animals selected for elimination were in poor health, suffering from forelimb lameness, posthitis, or showed poor general health status. All tested animals showed no abnormalities in pathological examination. In 2016, TB was confirmed in one individual (male, ca. 13 years old). The animal did not demonstrate any particular clinical symptoms, and in the cover letter was only recorded as being in poor condition. No lesions, including those suggestive of tuberculosis, were observed during anatomopathological examination. Bacteriological culture and molecular analysis revealed the presence of *Mycobacterium bovis* spoligotype SB2680 (not registered in the Mbovis.org databases and the Spol DB4 database).

The first case of TB in this area was reported in 2013 in the Wolisko European bison Breeding Center, part of the Borecka Forest. TB associated with *Mycobacterium caprae* spoligotype SB1912 was disclosed in one male wisent. The animal was around four years old and in generally good condition. After immobilization, the comparative intrapalpebral TST was performed with bovine tuberculin purified protein derivative (bPPD) (Bovitubal 28000, Bioveta, Ivanovice na Hané, Czech Republic), as previously described by Didkowska et al. [22]. A mild swelling of the upper eyelid was seen, and this was considered a positive result. No lesions suggestive of tuberculosis were observed during anatomopathological examination. It was likely that the animal was already infected when brought from the Smardzewice herd in central Poland: a herd with a known history of tuberculosis [23]. Therefore, this case probably had nothing to do with any previous case of tuberculosis in this area.

The European bison has been inhabiting the Borecka Forest since 1956 when scientists from the Polish Academy of Sciences introduced the species to a closed breeding herd. In 1977, during the renovation of the fence, a herd of bison escaped into the forest and some of the animals continue to live in the wild. The Borecka Forest occupies an area of 250 km^2^, together with many dairy cattle pastures in the neighboring area, along the border with the forest. It is likely that this is the first case of interspecies transmission between the local cattle and the European bison: documentation archived since 2011 revealed no cases of TB in cattle in the counties adjacent to the forest border. In 2016, immediately after confirming the occurrence of TB in the European bison, the Veterinary Officer ordered an annual tuberculin test of 1/3 of the cattle population in the voivodship; in other Polish voivodships, 1/5 of the cattle population is tested annually. In 2021, five years had passed since the positive result in wisent and no case of TB had been found during this time. As such, the previous cattle testing scheme was restored.

The risk of interspecies transmission is highlighted by previous studies, indicating that cross-infection is possible whenever different animal species share a single pasture; indeed, mycobacteria can persist for even several months in shaded environments and in wetland [24,25]. 

Due to logical challenges and lack of any credible ante-mortem diagnostics, reliable diagnoses of animal tuberculosis in wildlife are often only possible post-mortem [26,27]. Although most of the results obtained from the culled animals in the present study were TB-negative, there remains a need for constant monitoring of the health of the European bison, and other wildlife, within the Borecka Forest. The forest is inhabited by small predators such as foxes, raccoon dogs, badgers, pine martens, polecats, and muskrats, as well as larger ones, such as wolves and lynxes. They are also accompanied by deer and wild boar, all of which are very susceptible to TB infection [28,29,30,31,32,33,34]. 

The *Mycobacterium avium* subsp. *hominisuis* (MAH) isolate obtained in the European bison is a member of the *Mycobacterium avium* (MAC) complex, and hence has zoonotic significance: all MAC members have a worldwide distribution and zoonotic potential [35]. MAH causes tuberculosis in birds and is also responsible for infections in swine, rabbits, and wild animals [35,36,37,38]. Furthermore, despite a generally decreasing incidence of TB globally, MAH is emerging as an important pathogen in pulmonary diseases in humans [39]. The increased incidence of nontuberculous mycobacterial (NTM) infections in humans has been attributed to an increase in other chronic lung diseases, the use of immunosuppressive drugs, and the circulation of pathogenic mycobacteria in the environment [35,40]. 

Mycobacteria differ from other bacteria due their cell wall consisting of a significant percentage of waxes, which bestows resistance to many external factors. This feature is extremely important in the case of disinfection. The most effective tuberculocidal preparations are preparations that contain inter alia peracetic acid, hydrogen peroxide, ethanol, 2-propanol, sodium hypochlorite, chloramine, formaldehyde, or glutaraldehyde in appropriate concentrations [41].

Infection with the *M. avium* bacillus can complicate the intravital diagnosis of tuberculosis in cattle and result in the occurrence of false positive results in the tuberculin skin test (TST).

Since 2009, in accordance with EU requirements, Poland has been recognized as a country officially free from animal tuberculosis, i.e., with a prevalence below 0.1% of cattle herds, and the emergence of a new outbreak of TB in free-ranging European bison may negatively affect the maintenance of such status. In addition, there are problems with the interpretation of the Animal Health Law (AHL) in relation to the “king of the forest”, the European bison. Regulation 2016/429 applies in the territory of the Republic of Poland, as in the territories of all other European Union countries [42]. In some areas, new EU rules have been prepared that introduce changes to the protection of animal health, one being the division of infectious animal diseases into five categories: A to E. The AHL defines animal tuberculosis as being caused by *Mycobacterium tuberculosis* (human mycobacterium) and two bovine mycobacteria *Mycobacterium bovis* and *Mycobacterium caprae* cause tuberculosis and are subject to mandatory eradication. This particularly dangerous bacterial disease is classified as category B, D, and E only in bovids (*Bison* spp., *Bos* spp., *Bubalus* spp.). It is also important to note that such eradication applies to *Bison* spp. which is listed as susceptible to tuberculosis infection (*Bison bonasus*). Until 2021, the control of animal tuberculosis was specified by the general Polish provisions contained in the previous Protection Of Animal Health and Combating Infectious Diseases of Animals Act [43]. The AHL introduces a more universal, albeit very general, division of all animals into kept animals and wild animals. European bison kept in a pen fall within the definition of kept animals and are considered for the definition of “cattle”. These regulations indicate that in terms of movement between EU Member States, such bison should be treated as kept cattle. As such, they are subject to the health requirements for cattle, which are high. If bison kept in pens were to be treated as kept cattle they would also have to be subject to requirements regarding the identification and registration of cattle. Hence, it would appear that in the current legal environment, combating tuberculosis in European bison will depend on how they are kept. In the case of bison kept in pens, these will be determined by the rules set out for cattle, while for wild animals, no measures will apply.

Despite some inconsistencies, the introduction of the AHL helped to clear legal obstacles to the eradication of animal tuberculosis in European bison. This is very important also in the context of the threat of other infectious diseases. However, it is important to caution that bison herds become at high risk of disease, due to their low genetic diversity, which makes them susceptible to pandemics [4]. Worth mentioning that in the current legal system, there is no doubt that District Veterinary Officers in Poland have the competence to control and prevent animal tuberculosis in European bison.

## 5. Conclusions

Our obtained results demonstrate the importance of the monitoring of bacteria from MTBC in European bison in Poland.

## Figures and Tables

**Table 1:** Starter sequences were used in the reaction.

Identification	Target DNA	Primer	Sequence (5′–3′) *	Sequence Length (bp)
*Mycobacterium* genre	16 S rRNA	Mycgen–F	AGA GTT TGA TCC TGG CTC AG	1030
		Mycgen–R	TGC ACA CAG GCC ACA AGG GA	
*Mycobacterium tuberculosis* complex	MPB70	TB1–F	GAA CAA TCC GGA GTT GAC AA	372
		TB1–R	AGC ACG CTG TCA ATC ATG TA	

* The primer sequences were obtained from the European Reference Laboratory for Bovine Tuberculosis in Madrid.

**Table 2:** Reaction components.

Reagent	Concentration	Volume (µL)
Nuclease-free water	-	3.4
Multiplex PCR Master Mix	2x	12.5
MycF,R primers	35 ng/µL	1.5
TB1F,R primers	20 ng/µL	2.5
Mg^2+^	25 mM	0.1
DNA	-	5
Total volume	25	

**Table 3:** PCR Conditions.

Cycle	Temperature	Time
Initial denaturation	10 min	95 °C
Denaturation	30 s	95 °C
Annealing	2 min	65 °C
Elongation	3 min	72 °C
Final elongation	10 min	72 °C

## Data Availability

Not applicable.
